# Community-based behavioral health administrator perspectives on sustainability of Dialectical Behavior Therapy: a qualitative evaluation

**DOI:** 10.1186/s40479-020-0120-5

**Published:** 2020-02-28

**Authors:** Lauren B. Quetsch, Amy D. Herschell, Jane N. Kogan, James G. Gavin, Gaven Hale, Bradley D. Stein

**Affiliations:** 1 University of Arkansas, Fayetteville, AR USA; 2 Community Care Behavioral Health Organization, UPMC Insurance Services Division, Pittsburgh, USA; 3 UPMC Center for High-Value Health Care, UPMC Insurance Services Division, Pittsburgh, USA; 4 Otterbein University, Westerville, USA; 5 RAND Corporation, Pittsburgh, USA

**Keywords:** Dialectical behavior therapy (DBT), Qualitative feedback, Community behavioral health, Implementation, Sustainability

## Abstract

**Background:**

Substantial resources have been invested in evidence-based practice (EBP) implementation in community settings; however, research suggests that EBPs do not always sustain over time.

**Method:**

This qualitative study explored the perspectives of 13 community behavioral health agency leaders regarding the sustainability of an EBP 25 to 28 months following the original training period. Administrators from 10 agencies were interviewed to understand the complexities of the implementation process, sustainability of Dialectical Behavior Therapy, and their recommendations to enhance implementation and sustainability.

**Results:**

A content analysis revealed five emergent themes: treatment model opinions, resource concerns, staff selection/ turnover, population characteristics, and recommendations for future implementation.

**Conclusions:**

These themes likely would be helpful in informing the design of future implementation and sustainability initiatives sensitive to the challenges of integrating EBPs in community settings.

## Background

In an effort to enhance existing services, some behavioral health leaders have implemented evidence-based practices (EBPs) in community settings. Although agencies may be interested in adopting EBPs into their service line, implementation science is a relatively new field, and successful implementation strategies are not regularly reported in many EBP studies [1] thus limiting interested agencies’ capacities to recreate successful models or avoid implementation failures. Moreover, implementation efforts reportedly have been complex and sometimes difficult processes [2]. For example, competing priorities, uncertainty with job responsibilities, disagreements about agency goals and scale-up approaches, and poor fit with organizational cultures are a few of the reported barriers to successful EBP implementation [3].

Although there is still much to learn about components impacting EBP implementation, past implementation efforts have shown that one of the most important components to successful implementation is the involvement of administrative leadership [4]. Researchers concerned about getting EBPs from research laboratories to real-world community settings have recently focused on understanding community-based behavioral health (CBBH) agency leadership [2, 5] and how leadership factors (e.g., attitudes) or organizational level constructs (e.g., culture/climate) can influence the successful implementation of EBPs [6]. Even with a greater concern for understanding the unique challenges of CBBH agencies [7, 8], little information has been gathered directly from organizational leaders to inform implementation and sustainability efforts [9, 10]. Collecting data from these community-based leaders/experts about the challenges and successes of EBP implementation may likely increase the opportunity for an EBP to be sustained within practicing CBBH agencies [11].

Dialectical Behavior Therapy (DBT) is an EBP widely implemented and integrated into CBBH agencies across the United States [10, 12]. DBT is a highly effective treatment for individuals with borderline personality disorder (BPD) [13]; has been effectively adapted for other psychological disorders and problem behaviors including mood disorders, substance abuse, eating disorders, non-suicidal self-injury, intellectual disability, oppositional defiant disorder, and attention-deficit hyperactivity disorder [14]; and has been used with diverse populations (e.g., incarcerated individuals) [14]. BPD is characterized by impulsivity and instability in several areas of a person’s life, most notably relationships [15]. Often, these impulsive urges are displayed through self-injurious behaviors and extreme fluctuations in temperament [16]. Individuals with BPD are regularly characterized as difficult to work with [17], but DBT training has been found to improve clinicians’ attitudes towards individuals with BPD [12].

As an effective treatment for BPD and other presenting problems, many leaders of CBBH agencies are working to incorporate DBT into their practice [10, 18]. DBT is a multifaceted treatment involving four modes of delivery including individual therapy sessions, skills group training, weekly therapist consultation teams, and out-of-hours phone coaching by therapists to respond to client crises [19]. Without adequate awareness of the numerous factors impacting successful implementation of EBPs (e.g., funding, staffing resources, consumer concerns and how to address them, agency compatibility, system readiness [20]), efforts to implement EBPs, including DBT, may lead to failed treatment impact or long-term sustainability.

Previous literature has demonstrated the difficulty of sustaining some EBPs, including DBT, over time [10]; however, there is limited research to guide what does work in DBT implementation in CBBH settings (in which resources and infrastructure are often scarce). Efforts to integrate DBT into CBBH agencies present several challenges that limit the success of this EBP’s roll-out [21]. A common concern is the significant increase in demand placed on therapists who take on the role of conducting DBT in addition to their other responsibilities. Moreover, the necessary investment and reallocation of agency resources (e.g., staff commitments and time, funding, client recruitment) toward DBT implementation efforts [21] can also place a significant strain on agencies. It is essential to understand the challenges agencies face when trying to implement DBT, and also get a more comprehensive understanding why many DBT sustainability efforts are not as successful as would be ideal. Importantly, further investigation is warranted to determine how some agencies, despite these challenges, are successful in maintaining the treatment over time.

To tease apart the complex factors contributing to the success or failure of DBT sustainability, a qualitative approach is necessary. Qualitative research allows for the ability to examine issues in depth through open-ended responses from expert reporters in the field [22]. In turn, answers are not limited by numerical responses to a predetermined set of factors (e.g., Likert scale on assessment measure) and have room to shift directions and be explored further. Findings from CBBH agencies and leadership perspectives can help inform other CBBH agencies by collecting themes from leader interviews. Moreover, subtle factors that may greatly impact the effectiveness of implementation or sustainability within an agency may be unearthed in this format, while surface-level quantitative inquiries may miss these nuances. A qualitative approach was utilized in the current project to access the strengths of this methodology and inform future sustainability efforts for DBT in CBBH agencies, particularly considering Community Care’s investment in scaling and sustaining DBT.

### Previous evaluation

In 2009, Herschell and colleagues conduced qualitative interviews with agency administrators prior to the implementation of DBT in multiple CBBH agencies [23]. Findings indicated a need for understanding fit of agency structure for delivering services, resources necessary to successfully run an EBP, and how high rates of therapist turnover in behavioral health can impact service delivery [23]. While the literature highlights important components critical to EBP implementation and sustainability, sustainability of DBT requires further investigation due to the potential clash with an agency’s existing practices, increased therapist responsibility, securing a client base for steady DBT implementation, and high level of agency resources [23]. The present study is a follow-up to the Herschell et al.'s (2009) qualitative study which assessed administrator perspectives of DBT during the implementation process [23]. Outcomes in the earlier study indicated four major themes including opinions of DBT and its fit with agency practices, concerns with resources, the process of selecting therapists for DBT training, and the process for getting client referrals.

### Current study

In the present study, researchers examined CBBH leadership perspectives on DBT implementation and sustainability after training completion by conducting interviews with agency leaders (e.g., CBBH agency directors whose responsibilities included allocating agency resources, personnel staffing, managing adherence to federal/state/local regulations, supervision of clinicians) who engaged in a DBT implementation initiative [23]. Leaders were utilized as reporters due to their expertise in their agencies, communities, and service structure. More so than other possible reporters (e.g., clinicians), leaders’ knowledge was invaluable due to their breadth and depth of knowledge of the internal structure of the agency, population, and needs of the community and staff. Administrators were asked a series of questions by research staff related to the agency’s EBP sustainability and included the current status of implementation effort with each agency, feedback on the implementation process, feedback on the treatment model, and additional suggestions and future directions (Additional file 1). The objective of the current project was to assess agency administrators’ perspectives on DBT sustainability efforts and to understand how the process may be improved in future implementation efforts at CBBH agencies.

Aims of the study included determining what agency administrators would find challenging about DBT implementation even after significant efforts by trainers were made to prepare agency staff, and themes on both DBT and sustainability as they related to agency resources, staff turnover, staff responsibilities, and benefits and difficulties of implementing DBT [10, 21, 23]. Findings from this study will provide insight into feasibility and impact of DBT implementation and sustainability.

## Method

### Setting

Behavioral health directors from four Pennsylvania counties collaborated with a nonprofit behavioral health managed care company (Community Care Behavioral Health Organization [Community Care]) to implement DBT in 10 CBBH agencies. Community Care along with partner counties invited participating agencies to send clinicians to the DBT training. The agencies then selected their clinicians, and with clinicians’ permission, provided those individuals’ names and contact information to the research staff. The DBT training and implementation process was facilitated by Behavioral Tech, LLC. Training occurred over 18 months and utilized the Intensive Training Model developed and recommended by DBT experts [24].

### Participants

#### Agencies

Ten agencies in Pennsylvania were included in the parent study [12] to implement DBT within their agencies. In the present study to assess for sustainability efforts, only eight agencies were still providing DBT. These eight agencies provided behavioral health services (100%), were largely independent outpatient clinics (88%), and were generally large in capacity (e.g., multiple locations, greater than 15 employees; 88%).

#### Administrative leaders

Thirteen administrative leaders participated in the present study, representing eight agencies. Administrators were largely female (x = 10; 76.9%), Caucasian (x = 12; 92.3%), with master’s degrees (vs. bachelors, doctorate; x = 8; 61.5%), with education in counseling (vs. education, social work; x = 4; 30.8%). These administrators included eight original administrators from the parent study [12] (61.5%) as well as five new administrative leaders who obtained responsibility of overseeing the DBT programs through delegated roles. Therefore, two agencies that did not sustain DBT did not have administrators participate in the study while each remaining agency had at least one administrative leader discuss sustainability efforts.

### Procedure

#### Administrator interviews

For this study, the research team recruited agency administrators through email correspondence. Administrators were then contacted with a phone call to schedule a phone interview. Phone interviews were conducted around administrator’s schedules. Data collection included 13 interviews. Study activities were approved by the Institutional Review Board at the University of Pittsburgh.

#### DBT training

The Intensive Training Model included a 5 -day in-person training, 6 months of self-study, and was followed by another 5-day training. This first training was composed of lectures by two doctoral level clinical psychologists, videos, and role play exercises. The 6-month self-study included practice assignments as well as treating clients using DBT. After this period, the second 5-day training involved DBT teams receiving consultation on their clients, teams, and overall program. Consultation continued for 12 months following this training period. Overall, the entire course of training and consultation lasted 18 months. The 10 CBBH agencies oversaw a total of 64 clinicians (counseling, social work, psychology, nursing) who delivered DBT [12].

### Sustainability

Researchers in the current study aimed to include all original participating agencies in sustainability assessments regardless of their continued DBT implementation; however, agencies no longer implementing DBT declined participation, typically passively (i.e., did not respond to research staff calls or email contact). Of the 10 agencies trained in DBT, eight continued to provide DBT at the time of the interview, which was 25 to 28 months after starting the DBT training. The researchers explored agency factors that may have contributed to sustainability of DBT over time. Although common barriers such as size of the agency or rate of agency turnover did not appear to impact agency sustainability, due to the small sample size and heterogeneity of the agencies included in the study, definitive outcomes related to agency factors cannot be concluded.

### Data collection

A review of the implementation literature and the pre-implementation questionnaire [23] (e.g., qualitative interview with agency administrators regarding efforts to implement DBT, strengths and weaknesses of implementing the model in their agency) guided the content of the semi-structured interview. After an initial pool of questions was generated, stakeholders in the areas of DBT, Community Care, and CBBH administration modified the interview. Interview guide topics included: aspects of clinician involvement in DBT, clinician turnover, opinions on training, overall opinions of DBT implementation, and agency modifications to the DBT model. Examples of interview questions included, “What would you recommend to other administrators who are considering implementing DBT in the future?” and “Have you made any modifications to the model to adapt it to your setting? If so, what were the modifications?”

Trained interviewers collected interviews over the phone 7 to 10 months after the 18-month training, consultation, and implementation efforts were completed (i.e., 25 to 28 months following the initial training). Interview duration ranged from 30 to 65 min. All interviews were transcribed.

### Data analyses

A content analysis approach was taken to analyze the present data. All transcripts were independently coded using the data software, Atlas ti, by two coders [25] trained in qualitative methods [26]. A codebook was generated from interpreting the administrators’ responses [23, 27, 28] created in the initial research study and based on Ryan and Bernard’s [29] coding procedures; new codes were added in the current project based on additional categories which emerged in this set of interviews. Once coding was finalized, themes were drawn from the data, which were determined by presence of code clustering (i.e., quotes frequently paired together by coders) and frequency of codes appearing in the data [29]. A total of 15 codes were selected to address administrator comments. Overall, coder reliability was high across codes (κ. = .93).

## Results

Results of administrator interviews were considered together. Five themes emerged from the interviews: opinions of the DBT treatment model and implementation process, concerns related to agency resources, the selection and turnover of staff, characteristics of the targeted population, and recommendations for future implementation efforts. To illustrate the opinions across agencies, themes and sample quotes can be found in Table 1.

**Table 1:** Themes and Sample Quotes from Administrative Leaders on DBT Implementation and Sustainability

Themes				
Opinions of DBT Model & Implementation Process	Concerns with Agency Resources	Selection/ Turnover of Staff	Characteristics of Targeted Population	Recommendations for Future Implementation Efforts
“folks all agreed that DBT was effective and valuable.” “the team appreciated all of the training.” “figuring out scheduling and coverage was tough.” “the on-call phone contact scares therapists.” “[the] motivational component [was] helpful.” “outpatient and DBT are supportive and fit nicely together.” “[the] consistency is tough … It’s easier to get caught up in the chaos.”	“as an administrator, you have to realize that the training is resource–intensive [and that you] have to balance that out. We now are getting a higher reimbursement, which is helpful, but [we] didn’t get that for the first year.” “we had to get cell phones for them. You would need to change the job description [of the therapists] to include on-call time.” “we do get more insurance fees when we see clients.”	“You really have to select the right people. [They] have to be as committed to the model and agency as possible.” “have to have … appreciation for the importance of fidelity.” “can’t be married to a different theory and must be willing to change.” “you have to be a strong, assertive clinician.” “clinician apprehension … they are concerned about being adherent.” “clinicians have to be open-minded and willing to try anything.”	“Having clients accept responsibility for their own behavior is difficult.” “Initially groups were tough … clients [had] strong personalities and war stories, but there has been lots of change and personal development with those clients.” “we’re finding that we have had a few [client] hospitalizations.”	“need a clear cut plan and goal.” “[Therapists] would have liked more skills training and hands-on examples of how to implement skills training in depth sooner.” “Administrators should also know that it is a difficult population and expensive [treatment] to implement.”

### Theme 1: Opinions of the DBT model and implementation process

Administrators communicated primarily positive opinions of DBT, its implementation, and its effectiveness. Of the comments regarding opinions of DBT across reporters, 63.4% of those opinions had positive valence (vs. 36.6% negative). Administrators reported “folks all agreed that DBT was effective and valuable.” Other administrators commented on the specific components of DBT that they liked stating “skills group” and the “spacing of intensive training” were helpful.

Although intensive, administrators stated that the training “pays off in the long run” and that the “team appreciated all of the training.” Administrators also commented on the structure of the training saying that “it ended up being very helpful. We were able to accomplish a lot between trainings.” Though administrators also acknowledged, “figuring out scheduling and coverage was tough.”

Administrators communicated that “it is tough to implement DBT in our program.” Some claimed that the difficulty stemmed from “the structure and accountability [of DBT].” Another administrative leader stated that it was “too intensive, overwhelming, and intrusive.” Another had concerns about the client population and claimed “engagement is toughest,” and “DBT and the [client] population don’t come naturally.” Administrators stated their teams “had limited access to some behavioral techniques” and needed more skills training.

A few administrators had concerns with the requirement of DBT therapists to have phone availability after business hours. One administrator stated, “the on-call phone contact scares therapists” because it was “outside of their typical responsibilities.” They also stated certain DBT techniques were difficult: “Clients aren’t used to using diary cards and chain analysis,” and “[the] consistency is tough … It’s easier to get caught up in the chaos.”

### Theme 2: Agency resource concerns

The second theme that emerged was related to resource concerns. Generally, administrators emphasized how funding their programs would have been difficult without county and behavioral health managed care company support. They also had to readjust their service model to accommodate the new expectations of therapists. “From a business perspective, one of the most helpful parts was that the county reimbursed us for lost productivity, which allowed the clinicians to get trained without losing money.” Training was expensive to agencies because of the “unbillable time.” Some administrators stated, “as an administrator, you have to realize that the training is resource–intensive [and that you] have to balance that out. We now are getting a higher reimbursement, which is helpful, but [we] didn’t get that for the first year.” Administrative leaders reflected upon the benefit of increased rates from the training, “we do get more insurance fees when we see clients.”

As for the increased demand on clinicians to be on-call for their clients, one administrator said, “we aren’t at 100% fidelity to the model because of on-call challenges.” Another stated, “we had to get cell phones for them. You would need to change the job description [of the therapists] to include on-call time.”

### Theme 3: Staff selection and turnover

Administrator interviewees commonly stated that DBT-trained staff did leave the agency, but many believed this was not due to the training itself. “The training had nothing to do with people leaving the agency.” On the other hand, leaders also mentioned that some did not complete the training because “the training was too much” or “clinician apprehension … they are concerned about being adherent.”

Administrators also mentioned that specific clinician characteristics were necessary for ongoing implementation to be successful. One administrator stated, “You really have to select the right people. [They] have to be as committed to the model and agency as possible.” This administrator went on to say, “we had someone who went through the training and then wasn’t invested in the model … that was a lot of staff time.” Another administrator stated, “DBT helps keep you focused and some clinicians [struggled] with that.” Another leader remarked “clinicians have to be open-minded and willing to try anything.”

Administrators identified important clinician characteristics for success in DBT implementation. One administrator felt clinicians “have to have an academic background and appreciation for the importance of fidelity.” Flexibility was valued as well. Clinicians “can’t be married to a different theory and must be willing to change.” For those who were unsuccessful, one administrator noted, “those clinicians felt the population was too difficult to work with.”

### Theme 4: Population characteristics

A fourth theme that emerged from administrator interviews was the focus on targeted population characteristics. After working with the clients, administrators consistently noted the complex issues the population had and how that challenged the clinicians. “Clients are more difficult. The acknowledgement of previous behaviors is tough [for clients].” “Having clients accept responsibility for their own behavior is difficult.” One noted, “clients are resigned that their life will continue to be the same” and pointed that this can make the therapy challenging. Another administrative leader stated how the population is hard to manage because “we’re finding that we have had a few [client] hospitalizations” as they have patients work through “the tough painful part[s].” Administrators noted the challenge from working with those with BPD but recognized the rewards from implementing DBT. “Initially groups were tough … clients [had] strong personalities and war stories, but there has been lots of change and personal development with those clients.”

### Theme 5: Future implementation efforts

Administrators discussed lessons learned from implementation. They observed that in their role, they “need[ed] a clear cut plan and goal” and to be “clear with staff about expectations.” Others mirrored that sentiment by saying the biggest factor was “to know the details ahead of time.”

In addition, administrative leaders also focused on the resources necessary to keep a team going for DBT to be sustainable. They stated a decline in productivity expectations and “incentives … or awards” were necessary. One stated, “you should have training to be a [clinical] team leader” due to its added responsibilities of managing other clinicians and following up on all of the agency’s DBT clients. Administrators also had recommendations noting, “even though [therapists] speak weekly with DBT trainers, they would have appreciated even more training and an ongoing dialogue with other providers.” “[Therapists] would have liked more skills training and hands-on examples of how to implement skills training in depth sooner.”

One administrator recognized the challenges of the population. “Administrators should also know that it is a difficult population and expensive [treatment] to implement.” Another noted the importance of spending time on “the commitment process” with the clients and that this should be communicated early.

Lastly, one administrator had more wide-reaching recommendations. “I would like to see DBT used more routinely. I’ve seen how devastating this disorder can be for people.”

## Discussion

The purpose of this study was to understand leadership perspectives on DBT implementation and sustainability after training completion to inform agency administrators looking to implement DBT within their community agencies. The study explored both consistencies and changes in leadership perspectives over time [23]. In the prior evaluation, perspectives focused on the fit of the treatment within the existing services provided at the agency (e.g., integrating DBT in to the agencies’ clinic structure and population, providing adequate resources to administer the model, reducing personnel concerns through careful selection of clinicians to be trained [23]). Administrators in the present study communicated barriers related to the intensive training structure of DBT, the model’s demanding requirements, and the challenges related to the target population. Three themes that remained consistent pre- and post-implementation were: administrators’ (generally positive) opinions of DBT, concerns related to agency resources, and staff selection and turnover. Implications and applications of administrative leader perspectives are discussed below.

### Evaluate for goodness of fit

Prior interviews indicated some agencies’ concern related to the goodness of fit between DBT and the agency’s current practices [23]. Agencies facing the greatest difficulties with integrating DBT within their programming structure ultimately abandoned the implementation of the EBP, also known as “de-adoption” [30]. Unfortunately, these administrators denied participation in the present interviews, preventing researchers from further exploring barriers to sustainability within their agencies [31]. Although training and implementation efforts by agencies were voluntary, and research staff took strides to adequately prepare agencies for integrating DBT into their current practices, it is apparent that the leap for some agencies was too great. The following suggestions may help further prepare administrators wishing to adopt these new treatments into their agencies.

### Try to understand the demands of implementation

Administrators pre- and post-implementation commented on the increased demands required of DBT. For example, the DBT model requires each trained clinician to have a caseload of clients who are appropriate for individual DBT treatment (with weekly or twice-weekly sessions), conduct weekly group DBT sessions (with approximately eight clients) which are recommended to run 2 h at a time, participate in DBT supervision meetings with other DBT clinicians, and be available by phone at all hours and days of the week. It should be noted that while certain demands may be unique to DBT implementation, many EBPs require added duties that can make it difficult to integrate and maintain EBPs within CBBH agencies [3, 20]. These increased demands on clinicians’ time as well as the effect the change of treatment delivery has on the agency’s billing are important components for administrators to think carefully about to adequately assess for model feasibility [32–34].

In addition to understanding the increased demands placed on clinicians, administrative leaders need to consider the additional monitoring that may be necessary to ensure treatment fidelity of this EBP over time. While the current DBT intensive training implementation strategy did not include review of digital recordings, and also did not require consistent review of particular cases, therapy notes, or diary cards, integrating these strategies into agency practice should be done to avoid drift from the DBT model; however, these additional steps to monitor clinician adherence to treatment over time reduce clinicians’ billable hours within a clinic and should be considered when preparing for the implementation process. This current model included some consultation where clinical questions about cases were asked and answered to create a structurally adherent program without significant procedures to monitor therapist or session treatment adherence. This was done as a means to enhance feasibility, although it may have been at the cost of model fidelity.

Even with the reduced responsibilities compared to other EBPs that are highlighted above, the high level of accountability and fidelity required of DBT implementation reportedly was difficult for some agencies. Future agency leaders looking to implement DBT may benefit from understanding the significant difficulties associated with establishing strong methods for adhering to EBP protocol and ongoing monitoring of the quality of treatment delivery. Specifically, the process of delivering evidence-based quality care often requires key structural components to be in place [35] such as administrative support in the form of maintaining and training appropriate staff, effective management of agency funds, and providing appropriate time for staff to fulfill all roles and responsibilities [36]. If the structural components are not present, even a motivated clinical staff may not be able to sustain an EBP over time. If the structural components have been appropriately attended to, then key factors to enhance DBT success include interpersonal variables within organizations such as supervision, team cohesion, team communication, and team climate [6] as well as staff interest and expertise [8]. While the involved agencies in the present study communicated their interest in adopting DBT early on [23], some agencies were unable to start or maintain DBT implementation due to various organizational barriers. Additionally, the research team did not adequately assess if agencies had experience being trained in and successfully sustaining any EBPs prior to the start of the present study. Therefore, it is possible that some of the stated difficulties administrators reported about DBT are confounded with the difficulties of implementing an EBP, in general. Either way, it is important for administrators to think critically and problem-solve potential barriers within the agency prior to adopting an EBP (e.g., funding over time) to help improve the likelihood for long-term sustainability.

In addition to barriers with initial or sustained implementation of DBT, some interviewees indicated that their agencies started adapting the treatment model due to concerns about funding or staff responsibilities to address concerns the agencies thought they could not overcome. Adaptation of EBPs has been shown to be a common practice in CBBH agencies [8]. Frequently, agencies in this study reported limiting the availability of clinicians by removing the option for clients to call for skill support after clinic hours. While agencies may not initially plan to change the DBT model, adaptation may occur from the lack of infrastructure to handle the demands of treatment implementation. Adaptations may allow clinicians to devote greater efforts to delivering components that they believe are more likely to be effective in helping their patients and provide greater flexibility in choosing what components they think are likely to be most appropriate for a given patient. While this is appealing to many practicing therapists, this approach should be carefully considered. Past research has shown that adapting DBT may compromise treatment effectiveness and sustained DBT implementation [37]. This is especially significant as clinical practitioners wanting to provide high quality care for their clients may apply the model in ways that mistakenly remove core and influential components, either reducing positive client impact or creating detrimental client outcomes [38].

As recommended by one administrator, advanced training in how to supervise DBT might be helpful, as an appropriately trained supervisor may be better able to monitor fidelity. Administrative leaders also highlighted the importance of agency support (e.g., reduced expectations of clinicians, increased recognition for clinician and agency efforts) considering the increased demands of DBT, which enhanced morale and overall program success [39]. Planning ways to maintain quality implementation of EBPs (e.g., treatment adherence monitoring, built-in time for clinician paperwork, EBP funding) is an important step for long-term success and sustainability.

### Attempt to work through negative attitude and prejudice

Clinicians’ negative perspective on working with DBT clients was also a common concern before and after implementation. Overall goals of treatment implementation can be halted, and outcomes of clients can be negatively impacted if administrators and their clinical staff have low opinions of those who would otherwise benefit from EBPs [40]. Trainers and agencies should consider addressing the potential barrier of low therapist commitment to the client population. If administrators are committed to providing DBT to enhance patient care, gathering feedback prior to training on therapist negative attitudes or prejudices toward BPD or clients with BPD can help administrative leaders focus their efforts toward increasing engagement in working with this population. Motivational assessments could be conducted throughout training to determine if additional training time should be dedicated to garnering clinician buy-in. Importantly, clinicians’ opinions have been found to change over time to be more favorable toward clients with BPD once they undergo training [12]. Long-term benefits of DBT implementation as stated by community agencies include increased self-efficacy and compassion for practicing clinicians, a clinic’s ability to address unique symptoms, and greater levels of hope and functioning for their clients [8]. Even still, administrators willing to assess and address clinician engagement and bias toward BPD may benefit from increased clinician motivation, better client services, and a greater likelihood of DBT sustainability [41, 42].

### Get ready by harnessing adequate resources for implementation

Administrators noted the importance of sufficient resources not only with respect to training (pre-implementation [23]), but also to sustainable implementation (post-implementation). Many administrators stated that implementation would have been impossible without financial support provided by the behavioral health managed care company and the counties to offset costs associated with personnel attending training and providing DBT. This finding is consistent with the literature on sustainability of other EBPs [39]. Administrative leaders noted that the higher billing rates were beneficial so that their teams could dedicate time and energy to learning and implementing the treatment. This might not have been possible without a one-time infusion of funds from the county and the behavioral health managed care company to the provider organization that was used to offset decreased clinician productivity rates.

Financial considerations are an essential component of EBP sustainability [39] and have been frequently discussed in different EBP literature (e.g., EPIS [32], Getting To Outcomes [33], CFIR [34]). Administrators should spend adequate time researching the gains that may be obtained from investing in an EBP (e.g., better client outcomes, increased billing rate of therapists) as well as the costs (e.g., lost income during training periods). Specifically, administrators should set up meetings with potential trainers to understand the time commitment, training obligations, and details of daily treatment delivery to determine if the EBP is a financially viable and profitable investment. Prior to adopting DBT within an agency, administrators should consider expanding their knowledge of upfront costs, hidden costs, and a long-term funding stream to promote DBT sustainability [43].

### Consider preparing clinicians and your agency

Administrators highlighted concerns related to selecting competent clinicians to deliver DBT. Having staff with high levels of interest and expertise may help facilitate successful DBT implementation [8]. Yet, even with administrators’ careful selection, 45% of the original, trained therapists left their agencies during this period [12]. Turnover within agencies has been shown to be a predictor for deterioration of EBP over time [44]. And while DBT’s sustainability compares favorably with other EBPs, all models struggle with staff turnover; staff turnover negatively impacts sustainability [10]. Open communication between researchers, administrators, and clinicians about literature on therapist turnover and factors associated with retention in EBP may be beneficial to clinician selection [44]. For example, administrators believed that only senior staff with extensive clinical experience and high levels of flexibility would be successful, still some clinicians came to training with low opinions of the potential for DBT success. Yet clinicians who held lower opinions of DBT made substantial gains in their opinions throughout the training [12]. Interestingly, while some therapeutic factors such as therapeutic relationship [45] and therapeutic progress [46] have been shown to influence staff turnover, organizational factors such as low organizational support, staff morale, productivity, and organizational effectiveness [10, 47] along with financial burdens [48] may also lead to staff turnover. Moreover, the implementation of an EBP may increase the likelihood that turnover will occur [45]. Making sure the satisfaction of agency staff is high and the structure of the organization is stable prior to adopting an EBP may be just as, if not more important, than which staff are trained to deliver the new EBP [10]. Therefore, administrators may benefit from discussions with clinical staff about interest and commitment to an EBP. Moreover, assessing capacity of the agency and clinicians may be an important first step prior to moving forward with EBP adoption.

### Limitations

There were several limitations to the current study. First, including a larger number of agencies and administrators in the research study may have allowed for thematic saturation and reduced the possibility that particular themes remained unexplored in this particular CBBH setting. While administrator perspectives seem to represent a small sample of agencies, this sample size is common in studies utilizing qualitative leadership perspectives in clinical leaders (*N* = 15) [49], agency directors (*N* = 7) [50], and administrators (*N* = 16) [4]. Furthermore, previous research has concluded that as few as six to up to 12 interviews can produce thematic saturation in qualitative samples [51]. Importantly, these perspectives are often unexplored [9, 10] even though they may provide valuable insight into implementation efforts [2, 5].

Administrator response rate was an additional limitation to the outcomes of the present study. Only eight of the original administrators were included in the present evaluation (approximately 61.5%). It is unknown how the inclusion of all the original administrators might have impacted the present study’s findings. Moreover, due to two agencies failing to initiate DBT implementation and agency administrators disinterest in continuing the study, the research staff was unable to gather their commentary about sustainability. Insights on the explicit barriers to implementation and eventual de-adoption of DBT from agency administrators that were no longer implementing DBT would have been valuable.

Generalizability was an additional limitation of the current project. Perspectives were collected from eight out of ten agencies located in eastern Pennsylvania counties which may not reflect other sustainability efforts in more urban areas or other regions facing different challenges or restraints. Moreover, the perspective provided on behalf of each agency is limited by the fact that it represents only one or two people’s opinions.

In addition to adaptations some agencies utilized, limitations seen by administrative leaders in these Pennsylvania counties may have stemmed from possible clinician nonadherence to the model. Moreover, the strain on clinicians implementing a treatment with high risk and difficult clients may have been too great if they were not provided adequate supervisor support to guide and ensure adequate treatment adherence.

It is possible that administrator reports of some of the barriers to sustaining DBT within their agencies may have occurred regardless of the EBP implemented. In fact, more recent work conducted with DBT sustainability suggests that there are factors that make all EBP implementation difficult (e.g., workforce turnover); yet, DBT compares favorably to other EBPs in maintaining treatment fidelity and outcomes over time [52]. The researchers did not collect data to understand if agencies were successful in implementing other EBPs; therefore, outcomes are limited in understanding if all the present barriers were limited to DBT implementation or could be expected in the implementation of another EBP.

Results from this qualitative study reflect administrators’ perspectives, but may not have fully captured the reasons for successful or problematic DBT implementation (e.g., administrator beliefs for clinician termination vs. surveying clinicians about why they exited their agencies). The study may have benefited from collecting data via observational measures or real-time measurements throughout the course of DBT implementation, rather than the retrospective nature of the current administrator interviews. Future research studies may benefit from incorporating quantitative analyses to interpret additional factors that significantly impact implementation efforts.

## Conclusion

DBT is an important EBP that meaningfully serves individuals with BPD and other emotion regulation conditions. To ensure that DBT reaches a wider number of individuals in need, it is essential that trainings in EBPs occur and that researchers continue to monitor and solve concerns related to EBP sustainability. Therefore, this study contributes to understanding the key role administrators play in implementing and sustaining behavioral health EBPs in their agencies as well as important factors that should be considered prior to and during EBP implementation. Communicating with administrators about the process of implementing behavioral health initiatives highlights the importance of their expertise in sustaining these programs over time. Importantly, outcomes from this research suggest that agency administrators and researchers work together to improve behavioral health efforts by: assessing the agency’s motivation to implement an EBP with fidelity, helping understand the short- and long-term financial costs of implementing an EBP, realistically evaluating the resources necessary to implement an EBP and balance those with current staff responsibilities, and also establishing a model for support within each agency to promote sustainability over time. The researchers hope that these barriers are appreciated so that future efforts to establish and maintain use of evidence-based treatments in CBBH settings may have greater success in implementation and sustainability.

## Supplementary information

**Additional file 1.** Appendix A.

## Data Availability

The datasets generated and analyzed during the current study are not publically available to protect agency and subject confidentiality, but specific de-identified data are available from the corresponding author on reasonable request.

## Abbreviations

BPD: Borderline personality disorder
CBBH: Community-based behavioral health
DBT: Dialectical Behavior Therapy
EBP: Evidence-based practice
