# Supplementary material for: Community-based behavioral health administrator perspectives on sustainability of Dialectical Behavior Therapy: a qualitative evaluation
Source: Borderline Personal Disord Emot Dysregul. 2020 Feb 28;7:5. doi: 10.1186/s40479-020-0120-5 (PMC7047370; doi:10.1186/s40479-020-0120-5)
Supplement: Supplementary file 1 — Additional file 1. Appendix A. [file 40479_2020_120_MOESM1_ESM.docx]

Appendix A

1. Interview Date:
2. Start Time:
3. Name of person being interviewed:
4. Role at agency:
5. Agency Name:

**Interview Questions:**

1. **Current Status of Implementation Effort with Each Agency**

Are you currently implementing DBT at your agency?

Are the clinicians who were trained continuing to use DBT?

**PROBES:**

For those that are, what do you think was most helpful in helping to do so?

For those that aren’t, what do you think were some of the challenges that made the clinicians choose not to implement DBT?

How many clinicians that were trained stayed with your agency? How many left? Did the training have any impact on their decision to leave the agency?

1. **Feedback on the Implementation Process**

When you think about the training and implementation plan, what were the **most helpful** parts? Why?

When you think about the training and implementation plan, what were the **least helpful** parts? Why?

What would you recommend to other administrators who are considering using a similar training model and implementation plan in the future?

**PROBE:**

Would you suggest any changes in job responsibilities for those clinicians who will be implementing an EBP?

1. **Feedback on the Treatment Model**

When you think about DBT as a treatment model, what were the **easiest components** to implement? Why?

What were the most **difficult components** to implement? Why?

Have you made any modifications to the model to adapt it to your setting? If so, what were the modifications?

What would you recommend to other administrators who are considering implementing DBT in the future?

1. **Additional Suggestions/Future Directions**

Do you have any additional feedback or is there anything else we should keep in mind?

Would you be interested in participating in any future studies, particularly one focused on workforce issues and the costs associated with evidence-based treatment implementation?
